# Distraction of cyclists: how does it influence their risky behaviors and traffic crashes?

**DOI:** 10.7717/peerj.5616

**Published:** 2018-09-12

**Authors:** Sergio A. Useche, Francisco Alonso, Luis Montoro, Cristina Esteban

**Affiliations:** 1 Faculty of Psychology, University of Valencia, Valencia, Spain; 2 DATS-INTRAS, University of Valencia, Valencia, Spain; 3 FACTHUM.Lab-INTRAS, University of Valencia, Valencia, Spain

**Keywords:** Cycling, Bicyclists, Traffic injuries, Distractions, Risky behaviors, Traffic crashes, Public health

## Abstract

**Background:**

Undisputedly, traffic crashes constitute a public health concern whose impact and importance have been increasing during the past few decades. Specifically, road safety data have systematically shown how cyclists are highly vulnerable to suffering traffic crashes and severe injuries derived from them. Furthermore, although the empirical evidence is still very limited in this regard, in addition to other human factors involved in cycling crashes, distractions while cycling appear to be a major contributor to the road risk of cyclists.

**Objectives:**

The main objectives of this study were, first, to explore the prevalence and trends of cycling distractions within an international sample of bike users, and second, to determine the influence of such distractions on road crashes suffered by cyclists, simultaneously considering the explanatory role of risky behaviors (errors and traffic violations) as potentially mediating variables between cycling distractions and traffic crashes.

**Methods:**

For this cross-sectional study, we analyzed the data obtained from 1,064 cyclists—61.2% male and 38.8% female—from 20 different countries, who answered an on-line questionnaire on cycling-related features, habits, behaviors and accidents.

**Results:**

The prevalence of different cycling distractions oscillated between 34.7% and 83.6%. The most common distractions were those related to the behavior of other users, physical elements of the road, weather conditions and phone calls. Age trends and differences were also found, thus establishing a positive association between age and distractibility during cycling. Furthermore, the effect of distractions on traffic crashes of cyclists was significant when tested together with age, risk perception and risky behaviors on the road.

**Conclusion:**

The results of this study support the hypotheses that distractions have a major prevalence among bike users, and that they play a significant role in the prediction of the traffic crash rates of cyclists, through the mediation of risky behaviors.

## Introduction

Considering the wide importance of mobility and transportation as essential parts of the daily life of individuals, road safety constitutes a substantial element in the community’s welfare. At the same time, traffic crashes (bearing in mind their real consequences and features) represent a serious public health concern (WHO, 2004; WHO, 2015; Gopalakrishnan, 2012). For instance, more than 1.2 million people worldwide pass away every year as a consequence of traffic crashes, making road traffic injuries a major cause of death on a global scale (WHO, 2015; Bonilla-Escobar & Gutiérrez, 2014). In this regard, transportation dynamics are involved in a constant change and, nowadays, different alternative means of transportation are making us reconsider the role of road safety as a mere vehicle/infrastructure-related issue, increasing our awareness about the causes of accidents and their related intervention with the aim of preventing negative safety outcomes for road users, with basis on the study of human factors (Montoro et al., 2000; Cooper et al., 2009; Porter, 2011). Nevertheless, most of the available research on traffic safety has been based on motorized vehicles and their users only, without considering the many factors affecting the road safety and the health of those users who choose emerging sustainable means of transport such as bicycles, i.e., *cyclists*, whose health benefits have also been compared to their road safety-related risks, keeping in mind factors such as their high vulnerability to suffering severe injuries in case of accident (Hartog et al., 2010; Stipdonk & Reurings, 2012), and the generalized lack of coverage and effectiveness of road safety education strategies aimed at both cyclists and pedestrians (Elvik & Vaa, 2009; Twisk et al., 2014; Alonso et al., 2016).

To sum up, traffic crashes involving cyclists, especially during the past few years, have become a growing concern for public health agencies and road safety practitioners (Tin Tin, Woodward & Ameratunga, 2010; Boufous et al., 2011). To say it rather literally, the health and safety of cyclists lies in the balance of road dynamics currently present in most countries (Forjuoh, 2003; Buehler & Pucher, 2017). Also, considering the complexity of the cycling task, and the often-problematic interactions of bicycles with heavier vehicles (Haileyesus, Annest & Dellinger, 2007), other road users (Chaurand & Delhomme, 2013), and infrastructural conditions (Buehler & Pucher, 2017; Useche et al., 2018a), one of the factors essential to the cyclists’ welfare would be to keep their attention focused, during their journeys, on the sometimes-unpredictable risks, precautions and safety behaviors that bicycling demands. In other words, cyclists need not to get distracted while riding.

Overall, *road distraction*—in short, a deviation of attention, which shifts from tasks critical to safe driving, riding or walking, to another marginal activity—is an increasing and deadly threat to road safety (Stavrinos et al., 2013; Stimpson, Wilson & Muelleman, 2013), which has been mainly studied in motorized-vehicle drivers; but, bearing in mind the widely proven impairment that distractions cause to the behavioral performance (Cooper et al., 2009; Craik, 2014), they should be studied in the case of every type of road user (Macy et al., 2014; Overton et al., 2015). Considering the habitual overstimulation implied by the road environment—especially for what concerns the urban context—distractions not only constitute an everlastingly latent factor for road users, but they also have a proven association with the probability of being involved in a traffic crash (Oviedo-Trespalacios et al., 2017). Moreover, and bearing in mind the high physical vulnerability of cyclists during traffic crashes, distraction substantially increases the odds of suffering severe injuries or even death.

Considering statistics, cyclists represent around 7.8%–10% of all registered deaths on the road (Montoro et al., 2014; Puchades et al., 2017). In a study on North American cyclists, Wolfe et al. (2016) found that cycling distractions are more prevalent during certain hours of the day, especially around midday (40% of cyclists reporting distractions) and during morning hours (07:30 AM and 10:30 AM, with frequencies of 32.2% and 29.3%, respectively). Around 75% of fatal or serious crashes involving cyclists take place in urban areas, and approximately 10% of dead or injured cyclists are children (RoSPA, 2017). Regarding accidents in which cyclists were found to have primary responsibility, in a recent study the NZTA (2017) found that 21% of them were clearly distracted just prior to suffering the traffic crash. Moreover, in the case of Spain, the analysis of the cyclists’ crash reports (provided by traffic agents during their investigation on accidents) from the period 2008–2013 showed that up to 89.3% of the 25,439 traffic crashes suffered by cyclists involved cycling distractions as one of their concurrent causes, while other potential factors such as inexperience (8.2%), alcohol or drug consumption (5.1%), fatigue or sickness (0.1%), or inadequate speed (0.1%) had a minor impact in the causation of accidents (Montoro et al., 2014). Furthermore, during the last 10 years, according to what is registered in the official records (2007–2016), out of the total of 48,230 cyclists involved in traffic crashes in Spain, 1.36% (656) were fatal victims and, regarding non-fatal causalities, 11.87% (5,725) were injured but not hospitalized, and an alarming percentage of 86.77% (41,849) was hospitalized due to the severity of their injuries (DGT, 2017).

In addition to cycling distractions, and despite the fact that the statistics substantially vary from one country/study to another, there are many factors that, regardless of being associated or not with the active attention of cyclists, also have a major incidence on traffic crashes involving cyclists: overall, human errors are the main contributory factor of cyclist-involving crashes; about 71% of them are preceded by the error of one of the involved parties (RoSPA, 2017), and distractions have shown, in experimental tests, to cause about 17% of driving errors (McEvoy, Stevenson & Woodward, 2006). Other complementary and relevant factors to consider in order to explain traffic crashes involving cyclists are: the often problematic interaction with motor vehicles—strengthened by the lack of bike lanes and infrastructural developments for what concerns cyclists’ safety (Haileyesus, Annest & Dellinger, 2007; Bíl et al., 2016; Useche et al., 2018a), and deliberate risky behaviors (typically labeled as *traffic violations*), among which we can list inadequate speeding (Boufous et al., 2011; Helak et al., 2017), non-compliance with traffic signals (Prati et al., 2017; Vandenbulcke et al., 2009) and alcohol/drug use (Helak et al., 2017; Prati et al., 2017; Cripton et al., 2015). Another factor which is worth mentioning is that some of the many cyclist causalities (even those involving injuries) are usually under-reported, thus biasing the official statistics on the issue (RoSPA, 2017). Also, it is important to highlight that, although it has been proved that risk factors play a relevant role in the explanation of cyclists’ crashes, the involvement of cyclists in traffic crashes does not necessarily imply their subsequent culpability.

### Different types of distractions, one single problem

Distractions on the road may have different origins, dynamics and consequences (Neyens & Boyle, 2007; Neyens & Boyle, 2008). However, most of them have been related to the same result: traffic crashes potentially cause preventable injuries among the involved road users (Kahn et al., 2015; Llerena et al., 2015). In the first place, cycling while being distracted by technology, especially for what concerns the use of cellphones, headphones and navigators, constitutes a relevant factor whose impact on the road safety of cyclists has been demonstrated by empirical studies such as the one performed by Ethan et al. (2016), and Wolfe et al. (2016), showing that headphones and visual/tactile devices could be the most prevalent distractors related to bicycle using. Nevertheless, there are many potential distracting sources other than technological devices, which may potentially influence the adverse outcomes of road safety (Stimpson, Wilson & Muelleman, 2013). Decker et al. (2015) highlighted that external distractions, such as billboards on the roadsides and elements inherently designed to attract the attention of drivers (Dukic et al., 2013) affect between 6% to 9% of collisions among motor vehicles caused by road users’ distractions. Other relevant external sources of distraction are related to the behavior of other road users, that, as in the case of distracted pedestrians or drivers, may influence the attention of cyclists during the riding task, causing potential impairments in their cycling performance and thus explaining many operational errors, subsequently deriving in potential traffic crashes. Finally, weather conditions and road features may play a significant role in catching the attention of road users; in fact, some environmental conditions modulated by weather, such as darkness and low visibility, are associated with a higher degree of perceptual errors such as distraction and lack of attention (Jägerbrand & Sjöbergh, 2016; Oikawa et al., 2016), and many infrastructural factors, such as the presence of obstacles in the way, may imply a substantial decrease in the cycling performance and higher probabilities of being involved in a traffic crash (Useche et al., 2018a).

Also, it is known that, although cyclists are highly prone to experience distractions on the road, there are some mitigating factors that differentiate them from motor vehicle drivers: bicycles are usually exempt from some typical distractors present in heavier vehicles (e.g., audio systems, integrated navigators and other on-board units), and the absence of noise-isolation makes cyclists more prone to detect sonorous stimuli potentially present in the road environment. Furthermore, the average low speed at which cyclists circulate, often related to the high density of traffic in urban areas, constitutes a factor which reduces the objective risk of crash and injury. Also, the growing availability of cycling lanes in various countries constitutes a relevant mitigating factor which not only prevents cyclists from suffering cycling crashes, but also reduces their severity (Kaplan, Vavatsoulas & Prato, 2014; Harris et al., 2013).

For what concerns the existing empirical studies on road distractions, most of them agree on the fact that distraction may explain a large part of traffic crashes suffered by road users, and their results have been useful for the improvement of policy and decision making regarding traffic accidents and their associated positive outcomes for traffic safety. For instance, following the results obtained by Dukic et al. (2013) when testing the potential impairment caused by billboards in the drivers’ attention, the Swedish authorities decided to remove a large set of electronic billboards under testing phase, considering their proven distracting effect on drivers, and the subsequent hazardous outcomes for their safety. Also, in Spain, billboards on the sides of inter-urban roads were prohibited in 1998 as a way of reducing the negative effects of road distractions on the performance of drivers. Also, Stutts et al. (2003) raised the urgent need for a better management of all forms of distraction, in order to ensure the road safety of users. Furthermore, road safety education of cyclists and drivers, together with a successful integration of cycling into normal traffic patterns, might help decrease the morbidity and mortality on the road (Kiburz et al., 1986). To sum up, these have been our main practical motivations for developing the present study, focusing on the specific case of bicycle users.

### Objectives of the study

The main purposes of this study were: first, to explore the prevalence and trends of cycling distractions in an international sample of bike users, and second, to determine their influence on road crashes involving cyclists, simultaneously considering the explanatory role of risky behaviors (errors and traffic violations) as potentially mediating variables between cycling distractions and traffic crashes.

## Materials & Methods

### Sample

**Figure 1:**
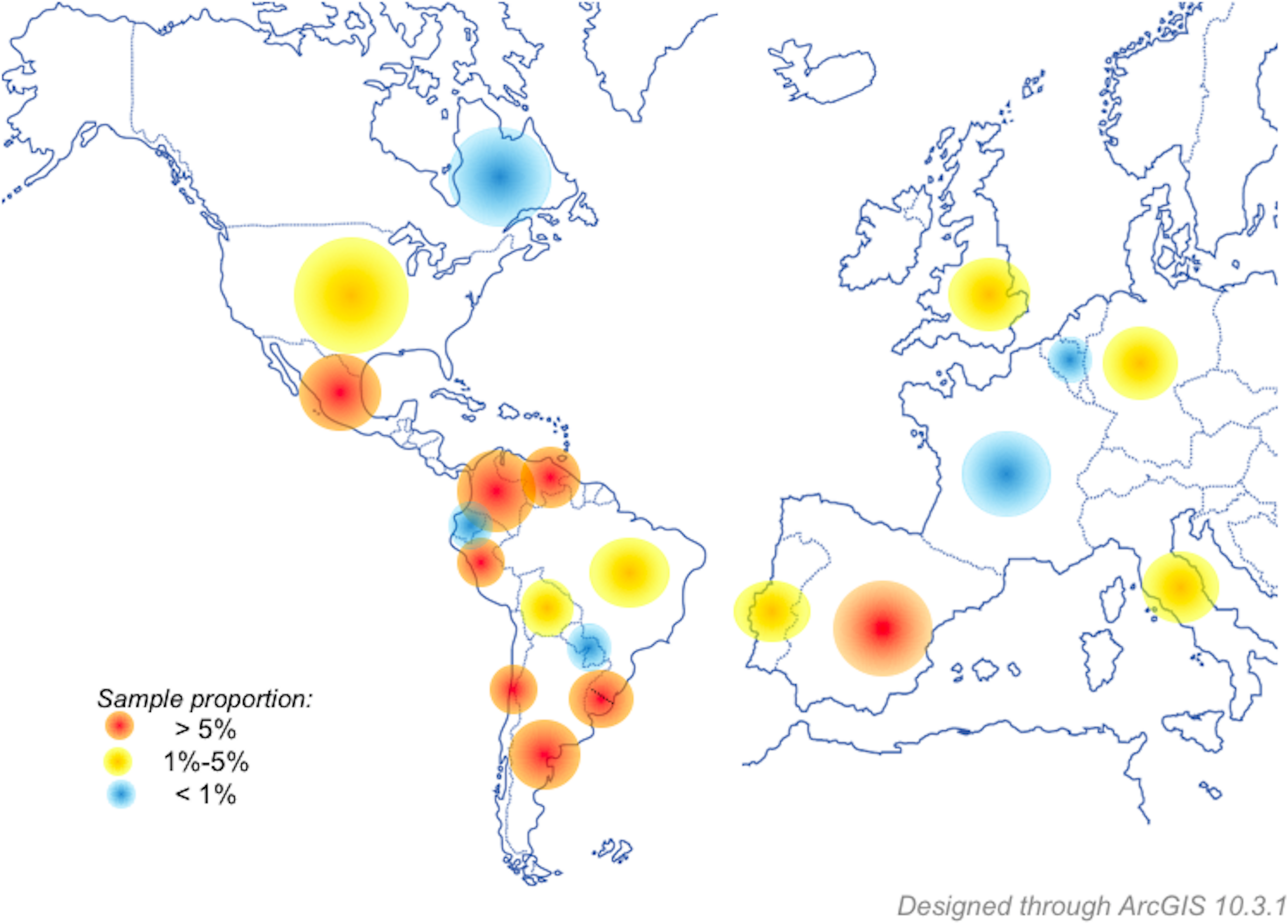
Geographical distribution of the sample. The countries/regions of provenance of the cyclists participating in this study. Differential colors indicate a greater (red) or lesser (blue) proportion of participants by country.

The data was obtained from a full sample of 1,064 bike users from 20 different Latin American countries (831 participants, representing 78.1% of the sample; 38.6% females and 61.4% males), Europe (161 participants, representing 15.15% of the sample; 41% female cyclists and 59% males), and North America (72 participants, representing 6.75% of the sample; 37.5% females and 61.1% males), 413 (38.8%) females, and 651 (61.2%) males. A graphic contextualization of the geographical coverage of the project can be seen in the Fig. 1.

Regarding the educational level of participants, approximately half of the respondents (50.1%) had an undergraduate degree, and 29.9% a post-graduate degree. A total of 9.1% of them had received a technical training (more advanced than a high school diploma, but lower than a university degree); 10.4% only had a high school diploma, and the remaining 0.5% had a maximum educational level of primary studies. They were aged between 17 and 80, with a mean value of *M* = 32.83 (*SD* = 12.63) years.

### Procedure

For this research, a convenience (non-probabilistic) sampling method was used, grounded on the availability and accessibility to the study population, and on their willingness to participate (or not) in the research. For this purpose, we designed an online (electronic) form in order to collect the data, and this was individually sent through an e-mail invitation to a wide sample of subjects contained in a pre-existent mailing list used for research purposes, composed of institutional members (staff, students and collaborators) and individuals who had previously participated in other studies.

The data collection was performed by the staff of the INTRAS-University of Valencia. During the presentation of the survey, potential participants accessing the online form were informed about the existing laws on data protection and about the fact that the collected information would only be used for statistical and research purposes. They were also asked, prior to the beginning of the electronic survey, to complete the questionnaire only if they were frequent bicycle users (it was suggested, as a guideline, “*if you ride a bike with certain regularity, at least once per week, or once every few weeks*”).

It is worth mentioning that, although this data collection method implies several advantages for the researchers, among which we find the increase of the efficiency, approachability and expediency of the data collection, the fact that subjects participating in this study belong to a pre-verified source, and that the questionnaire can be easily adapted to the time availability of participants (Tyrer & Heyman, 2016), it yet remains vulnerable to a reliability-related bias, given the impossibility to check, for instance, the veracity of the basic data (i.e., demographics) provided by the respondent (for further information, please see *Limitations of the study*).

### Description of the questionnaire

The questionnaire (Appendix I) was administrated in Spanish, and consisted of four sections: The first part asked about individual and demographic variables, such as age, gender, region of provenance, educational level and occupation.

As for the second part, self-reported risky cycling behaviors were assessed using the raw item bank of the Cyclist Behavior Questionnaire (CBQ) (Useche et al., 2018c), a self-report measure of road behaviors specifically designed for measuring high-risk riding behaviors (errors and violations) performed by cyclists. This Likert scale is originally composed of 44 items distributed along three factors: *Violations* (V), consisting of 16 items; *Errors* (E), composed of 16 items; and *Positive Behaviors* (PB), consisting of 12 items. A global score of *Risk Behaviors* was built through the sum of Errors and Violations reported by respondents.

The entire questionnaire used a frequency-based response scale of 5 levels: 0 = never; 1 = hardly ever; 2 = sometimes; 3 = frequently; 4 = almost always. Regarding the study’s factors, the first two dimensions were grounded on the original factorial structure of the Driving Behavior Questionnaire (DBQ) (Reason et al., 1990). Additionally, this scale includes a supplementary factor (positive of protective behaviors) for measuring behaviors that, unlike the first two dimensions, may help to prevent the occurrence of traffic accidents. To measure the risk perception and the knowledge of traffic regulations among cyclists, the Cyclist Risk Perception and Regulation Scale (RPRS) (Useche et al., 2018a) was used; it is a Likert scale composed of 12 items (seven for risk perception and five for assessing general rules of bike use), in which the degree of risk perceived in objective risk factors and the knowledge of general road regulations are assessed in a scale from 0 (no knowledge/risk perceived) to 4 (highest knowledge/risk perceived).

Thirdly, for assessing cycling distractions, an eight-item scale was build using dichotomous questions (yes/no), aimed at presenting different potential distractors and determining their self-reported presence and influence on the participants’ common journeys.

The last part of the questionnaire consisted of a series of questions related to the use of bikes, such as the average use of the bicycle (including average distances traveled and length of trips) and reasons for using it as a mode of transportation. Finally, this section of the questionnaire also included two questions about the traffic crashes suffered by participants, as cyclists: first, if they had suffered (or not) cycling crashes during the previous five years—regardless of their severity, but specifying that they were non-fatal crashes—and, second, in case of an affirmative answer, the number of crashes suffered during this period while cycling. In this sense, the variable *traffic crash rate* can be understood in this study as “the total amount of traffic accidents or crashes suffered while cycling during the last 5 years”.

### Ethics

In order to carry out this study, the Social Science in Health Research Ethics Committee of the University of Valencia was consulted. They certified that the research responded to the general ethical principles, was currently relevant for research in Social Sciences, and accorded with the Declaration of Helsinki, thus issuing a favorable opinion (IRB approval number H1517828884105). Furthermore, an informed consent statement containing ethical principles and data treatment details was used for all participants, explaining the objectives of the study, the average duration of the survey, the treatment of personal data and the voluntary participation, and it was always provided to the participants before they answered the questionnaire. Personal and/or confidential data were not used, and participation was anonymous, implying no potential risks for the integrity of our participants.

### Statistical analysis

In addition to the descriptive analyses, conducted in order to obtain the frequencies of distractions experienced by cyclists, and to the average scores for the used scales, a correlation analysis was performed to establish potential relationships among the variables of the study. After testing basic parameters and comparability criteria, a one-way ANOVA and Welch’s mean difference tests were used for comparing the average scores of distractions between gender and age groups. Furthermore, the association between distractions, age and risk perception, traffic crashes suffered while cycling during the previous five years, and the potential mediation of this relationship with cycling errors and normative violations, were tested using structural equation modeling (SEM). Weighted Least Square Mean and Variance Corrected (WLSMV) estimation was used, given that some data were ordinal and that the assumption of multivariate normality was not met (Caycho-Rodríguez et al., 2018; Finney & DiStefano, 2013). The model fit was evaluated through several statistics and indices from different logics and families. In this particular case, all available types of indices for the method of estimation were used: the chi-square, CFI, and RMSEA. Fit was decided based on the cut-off criteria expanded in the literature. A CFI above .90 (better if above .95) and RMSEA below .08 (Marsh, Hau & Wen, 2004) were indicative of an adequate model fit. Significance of parameters was established at *p* < .05. SEM were estimated in Mplus 8.0 (Muthén & Muthén, 2007–2018).

## Results

First of all, it is important to summarize some of our key findings on cycling habits and patterns and on the cyclists’ crash history found in the study sample. Regarding their journeys as cyclists, participants used their bicycles for a mean time of *M* = 6.71 (*SD* = 6.34) hours a week, being this the indicator of *hourly intensity* used in this study. The mean duration of their bicycle journeys was *M* = 47.5 (*SD* = 42.6) minutes. Finally, and regardless of the severity, 39.9% of them had suffered at least one traffic crash while cycling during the past 5 years, while 60.1% had not registered any cycling crash.

### Frequency analyses

Table 1 summarizes the first block of descriptive statistics (i.e., frequency analyses), in order to determine the prevalence of distracting sources in our sample of 1,064 participants. Overall, all the distractors had a relevant influence over cyclists, in a rage between 34.7% and 83.6%. Specifically, and based on the list contained in the table, the most frequently reported distracting sources which affected cyclists were the behaviors of other road users (83.6%), the presence of obstacles on the way (83.5%), and the current weather conditions (68.5%). On the other hand, the least reported distracting sources were: billboards (visual elements) with 34.7%, people that found them attractive (47.5%), and text messages or chats. It is worth mentioning that, although they are part of the same potentially distracting element (i.e., the cellphone), telephone calls (64.9%) represent a more reported distractor than text messages (46.4%).

**Table 1:** Descriptive data on cycling distractions. (A) shows the prevalence (frequencies and percentages) of different distractors on the road potentially affecting cyclists. (B) presents the number of reported distractions. Overall, the most prevalent rate by participant was to experience between four and five distractions while cycling (42.2% of the study sample) out of the eight presented in the instrument.

(A) Descriptive data on cycling distractions.				
Distracting source	Yes		No	
	Frequency	Percent	Frequency	Percent
01. Text messages or chats	494	46.4%	570	53.6%
02. Phone calls	691	64.9%	373	35.1%
03. Billboards	369	34.7%	695	65.3%
04. People that I find attractive	505	47.5%	559	52.5%
05. My own thoughts or concerns	586	55.1%	478	44.9%
06. Weather conditions	729	68.5%	335	31.5%
07. The behavior of other users of the road	890	83.6%	174	16.4%
08. The obstacles in the way	889	83.5%	175	16.5%

(B) Proportion of participants according to their rate of cycling distractions.		
Amount of distractions reported (scale 0–8)	Frequency	Percent
0	15	1.4%
1	23	2.2%
2	61	5.7%
3	130	12.2%
4	225	21.1%
5	225	21.1%
6	190	17.9%
7	106	10%
8	89	8,4%

As for the number of distracting sources reported by each participant, Table 1B shows that 60.1% (six of each 10) of cyclists commonly experience between four and six of the measured distracting factors during their cycling trips. Also, it shows that only 1.4% of them think they are not affected by any of these eight distracting sources.

### Distractions, gender and age groups

For what concerns the second block of descriptive analysis, a continuous variable was built (i.e., distractions while riding); it summarized, based on the previous list, the number of distracting sources which commonly affected participants. In this regard, both cyclists who consider that they do not experience any of the distractions when using the bicycle and cyclists that report being affected by all of them were found.

Overall, the mean value obtained for the variable was *M* = 4.84 (*SD* = 1.79) distractions experienced by users, in a scale between 0 (minimum) and 8 (maximum). As for gender comparisons, the results showed that men are the users who get more distracted (*M* = 4.91; *SD* = 1.86), compared to women (*M* = 4.73; *SD* = 1.67), although the mean difference (ANOVA: *F*_(1,1062)_ = 2.333; *p* = 0.127; Welch: *F*_(1,944.907)_ = 2.447; *p* = 0.118) is not statistically significant at level *p* <0.05, and for that reason this data should be interpreted only as an observed trend.

Furthermore, age comparisons were performed in order to determine the hypothesized differences in distractions among different age groups. For this reason, five different age intervals were created for conducting the comparative analysis, as summarized in Table 2. In this regard, the highest mean value of cycling distractions was reported by bike users belonging to the interval between 46–55 years of age (*M* = 5.09; *SD* = 1.98), and the lowest one was found in the case of cyclists under 26.

**Table 2:** Distraction mean scores according to age interval. The mean values on cycling distractions (sum), according to the age group of cyclists, distributed in 10-year intervals.

Age interval	*N*	Mean	Std. Dev.	Std. Error	95% CI		Min	Max
					Lower	Upper		
<26	390	4.677	1.59	0.080	4.52	4.83	0	8
26–35	318	4.701	1.74	0.098	4.51	4.89	0	8
36–45	160	4.869	2.07	0.164	4.55	5.19	0	8
46–55	119	5.092	1.98	0.181	4.73	5.45	0	8
>55	76	5.842	1.79	0.205	5.43	6.25	2	8
Total	1,063	4.843	1.80	0.055	4.73	4.95	0	8

**Notes.**

ANOVA (between groups): *F*_(4.1058)_ = 7.998; *p* < 0.001.

**Figure 2:**
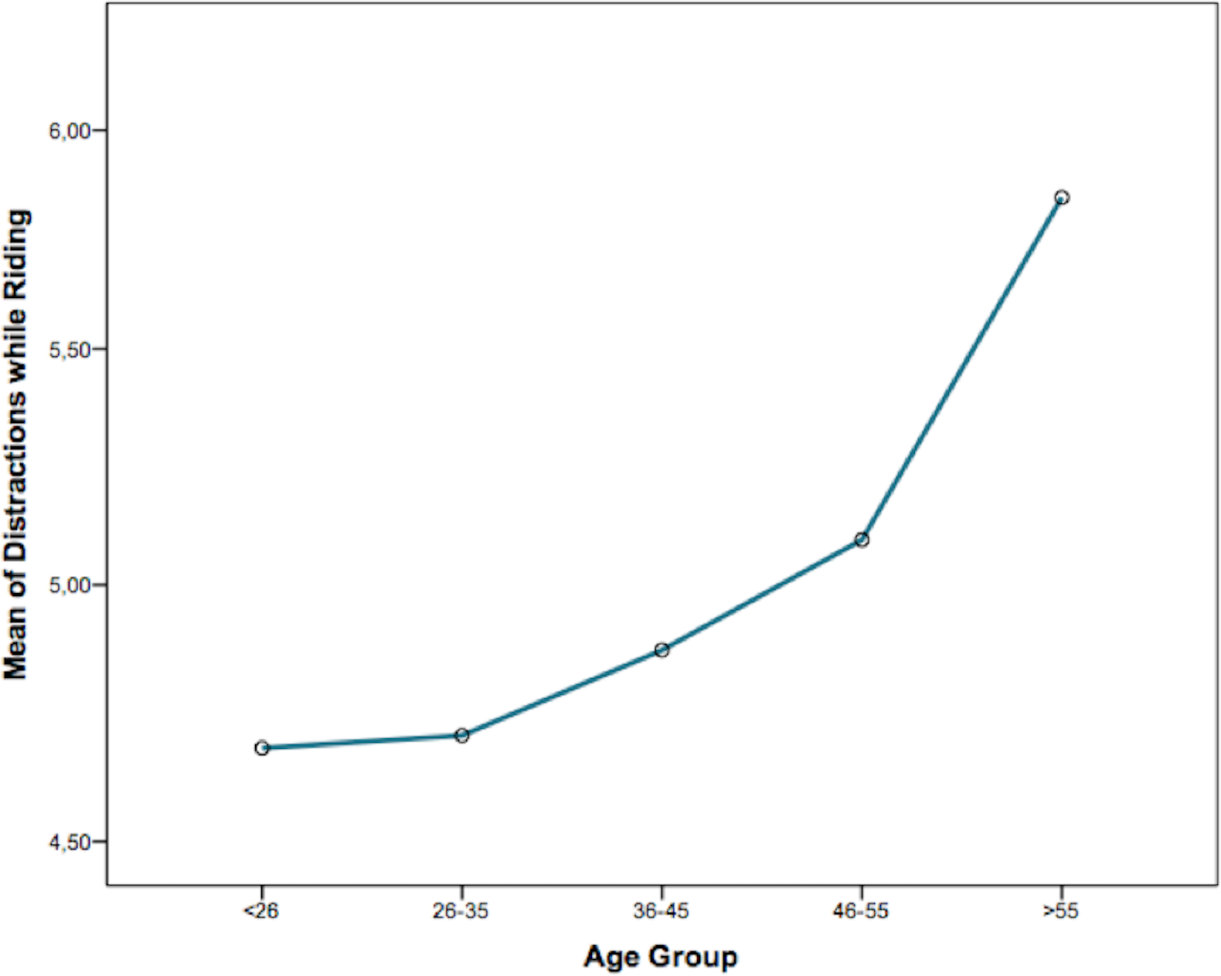
Mean of distractions while riding by age interval. (Comparatively) the average score on cycling distractions of each age group or interval. Overall, this value seems to be increased according to the age of cyclists.

Statistical analyses allowed us to find out that age is one variable differentiating distraction rates among bike users (*F*_(4,1058)_ = 7.998; *p* < 0.001), and we also observed a growing tendency in the scores they reported among the different age groups, as shown in Fig. 2.

### Correlation Analysis

The bivariate (Pearson) correlation analysis (see Table 3) for *σ* coefficients and significance levels permitted the identification of significant associations among cycling distractions, other individual factors, and traffic crash rates of the participants. Specifically, distractions were significantly related to cycling intensity (i.e., the average hours riding per week) [−], age [+], cycling errors [+], protective behaviors [−], and psychological distress of cyclists [+]. On the other hand, traffic crashes suffered during the last five years were significantly correlated to age [−], cycling intensity [+], cycling errors [+], traffic violations [+], level of knowledge of traffic rules [−], and psychological distress of users [+].

**Table 3:** Bivariate correlations between study variables. In (A), one can see the entire set of correlations between numerical variables of the study that arose from the analysis of a full participant sample (1,064 individuals). For (B), measures of association have been divided according to cyclists’ region of provenance. Although directions and significance levels are mostly coincidental a few differences can be observed, especially the relationship between demographic factors such as age and cycling habits. The association measure used (Pearson’s correlation coefficients) ranges between 0–1.

(A) Bivariate correlations between study variables (full sample)												
	Variable		1	2	3	4	5	6	7	8	9	10
1	Age	*σ*	1	−.177^**^	−.313^**^	−.146^**^	.173^**^	.362^**^	.244^**^	−.247^**^	.151^**^	−.197^**^
		*Sig. (2-tailed)*		0.000	0.000	0.000	0.000	0.000	0.000	0.000	0.000	0.000
		*N*	1,063	1,006	1,063	1,063	1,063	1,063	1,063	1,024	1,063	1,063
2	Hours riding per week	*σ*	−.177^**^	1	.293^**^	0.041	.116^**^	0.024	−.064^*^	−0.028	−.078^*^	.286^**^
		*Sig. (2-tailed)*	0.000		0.000	0.195	0.000	0.451	0.041	0.392	0.014	0.000
		*N*	1,006	1,007	1,007	1,007	1,007	1,007	1,007	969	1,007	1,007
3	Violations	*σ*	−.313^**^	.293^**^	1	.490^**^	−.307^**^	−.196^**^	−.241^**^	.140^**^	0.053	.361^**^
		*Sig. (2-tailed)*	0.000	0.000		0.000	0.000	0.000	0.000	0.000	0.081	0.000
		*N*	1,063	1,007	1,064	1,064	1,064	1,064	1,064	1,025	1,064	1,064
4	Errors	*σ*	−.146^**^	0.041	.490^**^	1	−.311^**^	−.290^**^	−.167^**^	.219^**^	.211^**^	.217^**^
		*Sig. (2-tailed)*	0.000	0.195	0.000		0.000	0.000	0.000	0.000	0.000	0.000
		*N*	1,063	1,007	1,064	1,064	1,064	1,064	1,064	1,025	1,064	1,064
5	Protective behaviors	*σ*	.173^**^	.116^**^	−.307^**^	−.311^**^	1	.382^**^	.326^**^	−.226^**^	−.064^*^	−0.011
		*Sig. (2-tailed)*	0.000	0.000	0.000	0.000		0.000	0.000	0.000	0.038	0.71
		*N*	1,063	1,007	1,064	1,064	1,064	1,064	1,064	1,025	1,064	1,064
6	Knowledge of traffic rules	*σ*	.362^**^	0.024	−.196^**^	−.290^**^	.382^**^	1	.350^**^	−.299^**^	−0.026	−.092^**^
		*Sig. (2-tailed)*	0.000	0.451	0.000	0.000	0.000		0.000	0.000	0.39	0.003
		*N*	1,063	1,007	1,064	1,064	1,064	1,064	1,064	1,025	1,064	1,064
7	Risk perception	*σ*	.244^**^	−.064^*^	−.241^**^	−.167^**^	.326^**^	.350^**^	1	−.158^**^	0.057	−0.049
		*Sig. (2-tailed)*	0.000	0.041	0.000	0.000	0.000	0.000		0.000	0.064	0.109
		*N*	1,063	1,007	1,064	1,064	1,064	1,064	1,064	1,025	1,064	1,064
8	Psychological distress	*σ*	−.247^**^	−0.028	.140^**^	.219^**^	−.226^**^	−.299^**^	−.158^**^	1	.086^**^	.065^*^
		*Sig. (2-tailed)*	0.000	0.392	0.000	0.000	0.000	0.000	0.000		0.006	0.038
		*N*	1,024	969	1,025	1,025	1,025	1,025	1,025	1,025	1,025	1,025
9	Distractions while riding	*σ*	.151^**^	−.078^*^	0.053	.211^**^	−.064^*^	−0.026	0.057	.086^**^	1	−0.025
		*Sig. (2-tailed)*	0.000	0.014	0.081	0.000	0.038	0.39	0.064	0.006		0.418
		*N*	1,063	1,007	1,064	1,064	1,064	1,064	1,064	1,025	1,064	1,064
10	Traffic crashes (last 5 years)	*σ*	−.197^**^	.286^**^	.361^**^	.217^**^	−0.011	−.092^**^	−0.049	.065^*^	−0.025	1
		*Sig. (2-tailed)*	0.000	0.000	0.000	0.000	0.71	0.003	0.109	0.038	0.418	
		*N*	1,063	1,007	1,064	1,064	1,064	1,064	1,064	1,025	1,064	1,064

(B) Bivariate correlations between study variables (sub-samples).												
		Region	1	2	3	4	5	6	7	8	9	10
1	Age	Latin America	1	−.166^**^	−.248^**^	−.114^**^	.160^**^	.282^**^	.217^**^	−.265^**^	.113^**^	−.143^**^
		Europe		−0.012	−.197^*^	−0.09	.221^**^	.279^**^	0.143	0.042	.198^*^	−0.057
		North America		−0.004	−.321^**^	−0.093	.317^**^	.291^*^	.303^**^	−0.229	.342^**^	−0.113
2	Hours riding per week	Latin America	−.166^**^	1	.283^**^	0.033	.110^**^	0.059	−.073^*^	−0.03	−.084^*^	.286^**^
		Europe	−0.012		0.151	−0.033	0.124	0.014	−0.027	−0.163	−0.053	0.137
		North America	−0.004		0.179	0.028	0.224	0.067	0.109	−0.095	−0.014	0.168
3	Violations	Latin America	−.248^**^	.283^**^	1	.467^**^	−.309^**^	−.115^**^	−.204^**^	.130^**^	0.059	.340^**^
		Europe	−.197^*^	0.151		.416^**^	−.262^**^	−.302^**^	−0.117	0.035	0.042	.299^**^
		North America	−.321^**^	0.179		.428^**^	−.335^**^	−.236^*^	−.250^*^	0.116	−0.14	.516^**^
4	Errors	Latin America	−.114^**^	0.033	.467^**^	1	−.318^**^	−.255^**^	−.164^**^	.215^**^	.230^**^	.216^**^
		Europe	−0.09	−0.033	.416^**^		−.214^**^	−.340^**^	−0.035	.228^**^	.207^**^	.232^**^
		North America	−0.093	0.028	.428^**^		−.431^**^	−.415^**^	−.296^*^	0.104	0.109	−0.025
5	Protective behaviors	Latin America	.160^**^	.110^**^	−.309^**^	−.318^**^	1	.401^**^	.315^**^	−.240^**^	−.093^**^	−0.001
		Europe	.221^**^	0.124	−.262^**^	−.214^**^		.253^**^	.347^**^	−.226^**^	0.002	−0.041
		North America	.317^**^	0.224	−.335^**^	−.431^**^		.479^**^	.373^**^	−0.01	0.05	−0.073
6	Knowledge of traffic rules	Latin America	.282^**^	0.059	−.115^**^	−.255^**^	.401^**^	1	.332^**^	−.297^**^	−0.046	−0.044
		Europe	.279^**^	0.014	−.302^**^	−.340^**^	.253^**^		.227^**^	−.225^**^	−0.086	−0.119
		North America	.291^*^	0.067	−.236^*^	−.415^**^	.479^**^		.523^**^	−0.114	0.109	−0.079
7	Risk perception	Latin America	.217^**^	−.073^*^	−.204^**^	−.164^**^	.315^**^	.332^**^	1	−.151^**^	0.026	−0.026
		Europe	0.143	−0.027	−0.117	−0.035	.347^**^	.227^**^		−0.083	0.115	−0.084
		North America	.303^**^	0.109	−.250^*^	−.296^*^	.373^**^	.523^**^		−0.174	0.173	0.001
8	Psychological distress	Latin America	−.265^**^	−0.03	.130^**^	.215^**^	−.240^**^	−.297^**^	−.151^**^	1	.084^*^	0.058
		Europe	0.042	−0.163	0.035	.228^**^	−.226^**^	−.225^**^	−0.083		0.103	−0.061
		North America	−0.229	−0.095	0.116	0.104	−0.01	−0.114	−0.174		0.185	−0.011
9	Distractions while riding	Latin America	.113^**^	−.084^*^	0.059	.230^**^	−.093^**^	−0.046	0.026	.084^*^	1	−0.017
		Europe	.198^*^	−0.053	0.042	.207^**^	0.002	−0.086	0.115	0.103		0.062
		North America	.342^**^	−0.014	−0.14	0.109	0.05	0.109	0.173	0.185		−0.173
10	Traffic crashes (Last 5 Years)	Latin America	−.143^**^	.286^**^	.340^**^	.216^**^	−0.001	−0.044	−0.026	0.058	−0.017	1
		Europe	−0.057	0.137	.299^**^	.232^**^	−0.041	−0.119	−0.084	−0.061	0.062	
		North America	−0.113	0.168	.516^**^	−0.025	−0.073	−0.079	0.001	−0.011	−0.173	

**Notes.**

*n* = 1,064.

*Sub-samples:* Latin America (*n* = 831), Europe (*n* = 161), North America (*n* = 72).

** Correlation is significant at the 0.01 level (2-tailed).

* Correlation is significant at the 0.05 level (2-tailed).

A specific correlation analysis based on the region of provenance of participants allowed us to confirm the high similarity in the directions of the association measures (Pearson’ coefficients) between study variables, when considering three subsamples (i.e., Latin America, Europe and North America). It is interesting to note how traffic crash rates of Latin American participants show differential values of significance if compared to cyclists from Europe and North America in the case of demographic (age) and cycling-related factors (cycling intensity). Despite the initial disproportionality of sub-samples used for this complementary analysis, most of the significance levels and magnitudes keep similar values, as shown in Table 3B.

### Structural equation modelling

**Figure 3:**
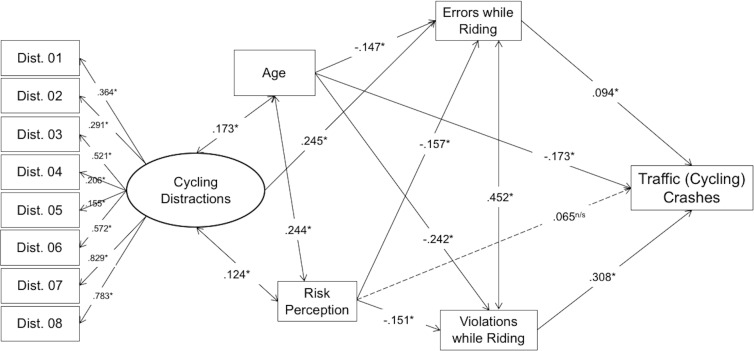
Structural equation model for predicting traffic crash rates. The directions and significances of the variables contained in the path (SEM) analysis. Both cycling errors and violations mediate the predictive role of distractions on traffic crash rates.

With the aim of testing the hypothesis of the effect of cycling distractions, age and risk perception in traffic crashes involving cyclists, and the potential mediating role of errors and traffic violations within an explicative dynamic, a structural equation model (SEM) was built according to the empirical directions reported in the introduction. Age, distractions (built up through the sum of an eight-item scale, as shown in Fig. 3) and risk perception were independent variables, violations and errors were mediators and the final outcome was the traffic crash rates. This initial completely a priori model did not fit the data well: *χ*^2^(55) = 746.46, *p* < .001; CFI = .810; RMSEA = .109, 90% CI [.102–.116]. Therefore, several modifications were made. Firstly, two non-significant and very low paths from distractions to traffic crash rates and to violations were set to zero. Secondly, a very large modification index that pointed out a relevant relationship between the two first indicators of distractions was included. With these three modifications, that made the model even more parsimonious, the model fit resulted adequate: *χ*^2^(56) = 408.83, *p* < .001; CFI = .903; RMSEA = .077, 90% CI [.070–.084]. All standardized parameter estimates are presented in Fig. 3, in which the unidirectional arrows indicate the direction of the predictive relationship, and bidirectional ones the correlational association between study variables.

In this regard, errors while riding (*β* = .094; *p* < . 05) and traffic violations (*β* = .308; *p* < .05) have a direct positive effect on the rate of traffic crashes suffered by cyclists (dependent variable). On the other hand, the explicative association of age (*β* =  − .173; *p* < .05) occurs in a negative direction, as observed in the unidirectional arrow linking age of cyclists and cycling crashes suffered. In other words, the higher the age of cyclists, the less prone to experience cycling accidents they are. As for risk perception (*β* = .065, *p* > .05), this variable showed a statistically non-significant explanatory effect on traffic crashes reported by cyclists, suggesting that it has not a direct influence on crashes.

Regarding the latter, and as for the hypothesized mediating role of errors and violations in the relationships among distractions, age, risk perception (independent variables) and traffic crashes (dependent variable), tested through this procedure, our results showed that errors while riding have significant relationships with age (*β* =  − .173; *p* < .05), risk perception (*β* =  − .157; *p* < .05), and distractions (*β* = .24; *p* < .05), as they are predictors of the dependent variable, i.e., the number of accidents suffered in the last five years, but essentially when they are linked to the commission of cycling errors.

As for the second mediating variable (traffic violations while cycling), this relationship follows the predictive model when considering age, i.e., more violations explain more traffic crashes (*β* =  − .242; *p* < .05), and risk perception (*β* =  − .151; *p* < .05). When indirect (mediation) effects were calculated together with traffic crash rates, the indirect effect of age was statistically significant in traffic crash rates (*β* =  − .088; *p* <  .05), as well as in risk perception (*β* =  − .061; *p* < .05), but not in distractions (*β* = .023; *p* > .05).

In other words, the observed relationships between study variables suggest that both errors and violations need to be kept in mind when considering the causal chain, in order to establish a predictive link between distractions and risk perception (independent variables), and the number of crashes suffered by cyclists during the last five years: a younger age and risk perception, and a higher score in cycling distractions predict more traffic crashes, through the commission of both non-deliberate (*errors*) and deliberate (*violations*) risky behaviors while riding.

## Discussion

Bearing in mind the first objective of the study, i.e., to explore the prevalence and trends of cycling distractions in an international sample of bike users, we hypothesized that road distractions affecting cyclists would present a high prevalence and a significant association with factors such as age and cycling behaviors.

Overall, the results of this research support the existence of a relationship between cycling distractions, individual variables, road behaviors and traffic crashes experienced by bicycle users. In this regard, and although the accumulated evidence on this subject (principally for what concerns the specific case of cyclists) is relatively limited, some key theoretical facts may enhance the comprehension of these relationships in the light of other empirical findings gathered in groups of cyclists from different countries. For instance, Montoro et al. (2014) found that most of the road crashes involving cyclists and causing, in many cases, considerable injuries and even death, were preceded by cycling distractions. Also, Wolfe et al. (2016) determined that distractions may differentially affect cyclists depending on the hour of the day, which is related to factors such as their motives to ride and the cycling intensity. Finally, Mwakalonge, White & Siuhi (2014) found that specific distractors, such as the use of electronic devices while cycling, constitute an unsafe behavior that could possibly be regulated by traffic rules, in order to avoid its potential incidence in risky road behaviors and crashes, whose odds are significantly increased by road distractions (Goldenbeld et al., 2012). However, traffic policies aimed at cyclists are notably scarce and remain a pending issue in most countries (Lang, 2007; Useche et al., 2018a), when they should instead be developed in the light of the specific demands and potentialities of every context, also considering the need of strengthening the development of sustainable and alternative transport modes (and the control of their associated risks), always framed within a responsible assessment of the impact of road safety strategies or measures, and their permanent challenges for the improvement of safety for cyclists and for all road users (Schepers et al., 2014; Schepers, 2013).

Summarizing the data, our first relevant point was the fact that, as for other groups of road users, distractions affect most cyclists with different frequencies and at different degrees. Thus, the answer to the question “*how prevalent distracted cycling behaviors are?*” seems to be relative and dependent on the types of potential distractors. In this sense:

- Distractions related to technological devices (i.e., cellphones, in the case of this study) may be determined between 46.4% (text messages/chats) and 64.9% (phone calls).
- Distractions related to visual elements (billboards) were the least prevalent within our study’s sample.
- People found attractive by cyclists—as a source of distraction—may imply a gender-related difference. Indeed, the differential frequency analysis showed that the prevalence among male cyclists was 62.1% (three out of five participants), and only 24.5% among females (one out of four).
- Intra-personal factors, in this case one’s own thoughts and concerns, affected 55% of the sample as a distractor.
- Weather conditions constituted a distractor for seven out of 10 cyclists.
- The (often problematic) behavior of other road users constituted the most prevalent distractor affecting cyclists (83.6%).
- Finally, the presence of obstacles on the road constituted a source of distraction for 83.5% of cyclists.

Moreover, distractions on the road presented significant associations with individual and psychosocial variables such as age, cycling intensity and psychological distress. Consistently with this, and keeping in mind the first hypothesis of the study, we found that the age of the subjects significantly differentiates the rates of distractions reported by road users, as it has been observed in other researches on drivers (Neyens & Boyle, 2008; Llerena et al., 2015), and cyclists (Goldenbeld et al., 2012). In this regard, our results showed that older individuals were the ones presenting a higher rate of distractors affecting their riding. Another age-related issue that is worth discussing is the fact that, although cycling distractions increase with age, traffic crash rates maintained a negative association with the age of cyclists. This phenomenon could be interpreted in the light of the results provided by some empirical studies, which reported that younger cyclists tend to present more risk-taking behaviors, and to have a higher risk of being involved in a traffic crash than older users (Martínez-Ruiz et al., 2014; Goldenbeld et al., 2012; Wardlaw, 2002). In other words, despite being less distracted while cycling, younger cyclists also tend to present higher values of risky behaviors, which may contribute to explain their higher involvement in traffic crashes compared to older cyclists. We will list some important trends found through the correlational analysis of our study: the younger the cyclist, the higher the scores in traffic violations and errors. On the other hand, scores in risk perception, rule knowledge and protective behaviors tend to be lower (for the entire set of correlations between age and other study variables, please see *Results*).

Regarding the second objective of the study (i.e., to determine the influence of road distractions on traffic crashes), we hypothesized that cycling distractions would predict both risky cycling behaviors and traffic crashes suffered by participants during the previous 5 years. In this sense, the structural equation model (SEM) built for predicting safety outcomes showed that, although distractions do not directly explain traffic crashes, they do influence them through the statistical mediation of both involuntary risky behaviors(errors) and intentionally committed behaviors (traffic violations), in the last case through the mediation of risk perception. Furthermore, there are no structural differences between errors and violations, as they predict the dependent variables (i.e., traffic crashes) in the same direction and mediating cycling distractions; thus, distractions are a key factor predicting road risky behaviors that, at the same time, and following the causal chain, also predicts traffic crashes. In this sense, some studies have agreed on the relationship between distractions and risky behaviors on the road, and on the importance of considering factors such age and cycling intensity as predictors of traffic incidents of road users (Stutts et al., 2003; Macy et al., 2014; Useche et al., 2018b). Finally, based on the study of smartphone-specific violations while cycling, Puchades et al. (2017) found results similar to the ones obtained in this study, but approaching near crashes as a potential mediator between errors, violations and crashes. Back to our study, the results obtained through the path analysis allowed us to confirm that road distractions predict the traffic crashes of cyclists, but only through the process of increasing risky behaviors, that play a mediating role in their predictive relationship.

Moreover, the results provided by this study allow us to remark the need of developing mechanisms and strategies aimed at reducing the distractibility of road users (Stutts et al., 2003), through interventions that are meant to enhance their interaction with infrastructural, technological and social factors that may strengthen road safety (Twisk et al., 2014), by means of a substantial reduction of risky behaviors when cycling, and (considering the high physical vulnerability of cyclists in case of an accident); these interventions should also develop the prevention of traffic injuries and human losses derived from—thanks to the growing scientific evidence in this regard—preventable road crashes. As for potential implications of transport policies, we have described in the introduction how some relevant findings provided by other studies have influenced the decision-making employed in the elaboration of road safety measures, such as the monitoring of both conventional and electronic billboards along certain types of roads (Dukic et al., 2013; Montoro et al., 2014) and the strengthening of the need to raise a social discussion on the management of cycling distractions for the improvement of traffic safety (Stutts et al., 2003). In this sense, and considering the proven relationship between cycling distractions, risky road behaviors and traffic crash rates of cyclists, and also the high prevalence of distractors related to problematic interactions with other road users, bad conditions on the road and mobile devices, this study remarks the need of strengthening road safety education and training both *for* and *towards* cyclists, in order to enhance: protective factors such as a better interactions among different road users and less risky behaviors among cyclists, based on their progressive and positive integration in the transport dynamics; a more responsible use of technological devices and the awareness of other potential distracting sources; and, finally, a substantial emphasis on the improvement of infrastructural conditions (i.e., more friendly cycling roads). Nevertheless, the aforementioned ideas constitute potential policy suggestions and measures that clearly exceed the scope of the sole study of cycling distractions; in other words, more information needs to be considered in order to determine the most accurate guidelines for decision making in road safety issues (Forjuoh, 2003; Vandenbulcke et al., 2009). This study represents, however, a first glance at the research of a typically underestimated and scarcely-studied topic of traffic crash causation among highly vulnerable road users, such as cyclists.

## Conclusions

Summarizing the main findings of this research, and keeping in mind the scope provided by the employed statistical analyses, this study made it possible to affirm that:

- Distractions on the road are a factor relatively common among cyclists, and, regarding the specific set of distractions we studied, their self-reported prevalence oscillates between 34.7% and 83.6%.
- An interesting trend was found in the prevalence of cyclists’ road distractions according to their age. Older individuals tended to be more affected by road distractions when cycling.
- Cycling distraction rates are also associated with personal variables such as psychological distress and the intensity of cycling, and with both risky and protective behaviors on the road.
- Also, cycling distractions play a significant role in the prediction of risky behaviors preceding traffic crashes involving bike users. In other words, distractions predicted the traffic crash rates of cyclists, but through the mediation of risky behaviors.
- Finally, this study suggests the need of examining the role of road distractions and other complementary factors both for what concerns cyclists and other road users, as a way to enhance the predictive ability and the global understanding of traffic crashes involving them.

### Limitations of the study and further research

Finally, some potential sources of bias and two essential facts related to the collection of data and to the analyses performed in this study should be mentioned. First, while this research employed a considerably large sample of cyclists, the survey was conducted through an electronic questionnaire and based on self-reported information.

Regarding this, and although research on individual differences in traffic safety—including most of the studies dealing with risky behaviors—has been developed mainly on the basis of self-reported data (af Wåhlberg & Dorn, 2015), it is worth mentioning the potential biasing effect of this data collection method on the reliability of our results. Specifically, some bias related to the use of self-report and to the convenience sampling, potentially affecting the results of the study, are: *response bias*, that may range from the social desirability (even when the questionnaire is anonymous, the tendency to show one’s “best look” in behaviors and attitudes may still be present, in this case related to road safety habits) to responses provided with a poor understanding of the questions or the survey dynamics (Rosenman, Tennekoon & Hill, 2011); these potential biases can be present even when instruments are carefully revised several times according to different ages and educational levels. Another bias may be the potential *under/overrepresentation* of the study’s population, that could make it difficult to perform further comparisons among subjects with (in this case) other habits for transportation; and, finally, it is worth mentioning the *selection bias*, i.e., the possibility that some population members were more likely to be asked to participate than others. For these reasons, it is important to remark the need for researchers to be careful when making generalizations based on the available data, since it may exceed the actual characteristics of the study’s sample.

And, also, we wish to remark that this is an international study, a commonly positive aspect which can nevertheless imply a negative side: the heterogeneity of traffic dynamics. In this regard, we should bear in mind that the specific traffic patterns and infrastructural advances in different countries may vary largely (Lahrmann et al., in press; Reynolds et al., 2009), factor which could be important when generalizing the results obtained for groups of cyclists that, although having similar individual characteristics, may be exposed to differential road risk factors modulating their road behavior. Specifically, and it is also important, the lack of data on other complementary factors (e.g., distractions from other road users, traffic volumes, average speed) could represent a major limitation for explaining a higher variance rate in the prediction of traffic crashes involving cyclists. For this reason, it is important to remark the need of considering multi-level designs and accident/crash related information (i.e., objective records) in order to enhance the predictive ability and external validity of the findings. Furthermore, it may be very useful to consider factors such as average speeds, the severity of reported cycling crashes and the traffic volumes through the Annual Average Daily Traffic (AADT) data—an important couple of missing element in this study—as categories to contrast the potentially differential impact of distractions on traffic crash outcomes of cyclists.

Regarding the SEM model fit, although the coefficients of the final model are good overall (CFI > .90; RMSEA < .80), the cutoff point for these coefficients may vary depending on the theoretical approach. Also, and although it was fulfilled in this research, the root mean square error of approximation (RMSEA) should not be pursued as the single way for determining the model fit (Chen et al., 2008). This is the reason why it is suggestible to consider the use of larger samples and the test of the model fit.

Finally, and speaking about further researches which may be conducted in this field, it is also suggestible to use samples with similar proportionalities in order to make potential comparisons between cyclist of different regions/countries, task that may contribute to obtain relevant information on the impact of cycling distractions within different contexts.

##  Supplemental Information

10.7717/peerj.5616/supp-1 Data S1 Raw data of the study The contained data set constitutes the raw data of the basic research. Main variables are already calculated using the proper method for each questionnaire.

10.7717/peerj.5616/supp-2 Supplemental Information 1 Blank questionnaire The blank form of the used questionnaire, including the analyzed sections. Please note that the original instrument was designed in Spanish, and this is a translation.

## Additional Information and Declarations

### Data Availability

The following information was supplied regarding data availability: The raw data are provided in a Supplemental File.
