# Supplementary material for: Distraction of cyclists: how does it influence their risky behaviors and traffic crashes?
Source: PeerJ. 2018 Sep 12;6:e5616. doi: 10.7717/peerj.5616 (PMC6139010; doi:10.7717/peerj.5616)
Supplement: Supplemental Information 1 — The blank form of the used questionnaire, including the analyzed sections. Please note that the original instrument was designed in Spanish, and this is a translation. [file peerj-06-5616-s002.docx]

**Appendix I. Questionnaire**

1. **Demographics**

**1.** **Gender:**    Man ☐     Woman ☐

**2.** **Age:** ___ years.

**3.** **What is your main occupation?**

☐ Student

☐ Employee

☐ Independent (Self-Employed)

☐ Unemployed

☐ Retired

☐ Housekeeping

☐ Other

**4.** **Highest educational level** (ongoing or completed):

☐ None

☐ Primary education

☐ Secondary Education - Baccalaureate

☐ Technical-intermediate training

☐ Undergraduate degree

☐ Post-graduate degree

**II.1 CBQ (Cycling Behavior Questionnaire) (44-item full version)**

Please estimate how often you do the following when cycling, using this scale:

*0 = Never; 1 = Almost never; 2 = Sometimes; 3 = Frequently; 4 = Almost always / always*

| **Item** | **Frequency Degree** | | | | |
| --- | --- | --- | --- | --- | --- |
| Despite having an exclusive bicycle lane nearby, I circulate on the vehicular lane or on the sidewalk. | 0 | 1 | 2 | 3 | 4 |
| Cycling under the influence of alcohol and / or other drugs or hallucinogens. | 0 | 1 | 2 | 3 | 4 |
| Circulating against the traffic (wrong way). | 0 | 1 | 2 | 3 | 4 |
| Listening to music while cycling. | 0 | 1 | 2 | 3 | 4 |
| Talking on the phone or texting while riding the bike. | 0 | 1 | 2 | 3 | 4 |
| Transiting on the left along mixed/vehicular lanes. | 0 | 1 | 2 | 3 | 4 |
| Zigzagging between vehicles when using a mixed lane. | 0 | 1 | 2 | 3 | 4 |
| Handling potentially obstructive objects while riding a bicycle (food, packs, cigarettes ...). | 0 | 1 | 2 | 3 | 4 |
| Feeling that sometimes I'm going at a higher speed than I should be going at. | 0 | 1 | 2 | 3 | 4 |
| Crossing what appears to be a clear crossing, even if the traffic light is red. | 0 | 1 | 2 | 3 | 4 |
| Carrying a passenger on my bicycle without it being adapted for such a purpose. | 0 | 1 | 2 | 3 | 4 |
| Using the vehicular lane and sidewalk interchangeably, using at times the one that allows you to go faster. | 0 | 1 | 2 | 3 | 4 |
| Sometimes stopping at crosswalks, or at other places that obstruct pedestrian traffic. | 0 | 1 | 2 | 3 | 4 |
| Whistling, shouting and/or using the bicycle bell, so that the driver ahead will go faster. | 0 | 1 | 2 | 3 | 4 |
| Having a dispute in speed or "race" with another cyclist or driver. | 0 | 1 | 2 | 3 | 4 |
| Yelling, gesticulating and/or ringing the bell to express anger towards someone on the road. | 0 | 1 | 2 | 3 | 4 |
| Unintentionally crossing the street without looking properly, thus making another vehicle brake to avoid a crash. | 0 | 1 | 2 | 3 | 4 |
| Colliding (or being close to it) with a pedestrian or another cyclist while cycling distractedly. | 0 | 1 | 2 | 3 | 4 |
| Forgetting the route or destination I was heading to. | 0 | 1 | 2 | 3 | 4 |
| Braking suddenly and being close to causing an accident. | 0 | 1 | 2 | 3 | 4 |
| Failing to notice the presence of pedestrians crossing the road when turning. | 0 | 1 | 2 | 3 | 4 |
| Not braking on a "Stop" or "Yield" sign, and being close to colliding with another vehicle or pedestrian. | 0 | 1 | 2 | 3 | 4 |
| Braking very abruptly on a slippery surface. | 0 | 1 | 2 | 3 | 4 |
| While I am distracted, I do not realize that a pedestrian intends to cross a crosswalk and do therefore I do not stop to let him or her do so. | 0 | 1 | 2 | 3 | 4 |
| Not realizing that a parked vehicle intends to leave and consequently having to brake abruptly to avoid a collision. | 0 | 1 | 2 | 3 | 4 |
| When driving on the right side, not realizing that a passenger is getting out of a vehicle or bus, and thus being close to hitting him or her. | 0 | 1 | 2 | 3 | 4 |
| Trying to overtake a vehicle that had previously used its indicators to signal that it was going to turn, consequently having to brake. | 0 | 1 | 2 | 3 | 4 |
| Misjudging a turn and hitting something on the road, or being close to losing balance (or falling). | 0 | 1 | 2 | 3 | 4 |
| Unintentionally, hitting a parked vehicle. | 0 | 1 | 2 | 3 | 4 |
| Failing to be aware of the road conditions and falling over a bump or hole. | 0 | 1 | 2 | 3 | 4 |
| Confusing one traffic signal with another, and maneuvering according to the latter. | 0 | 1 | 2 | 3 | 4 |
| Trying to brake but not being able to use the brakes properly due to poor hand positioning. | 0 | 1 | 2 | 3 | 4 |
| I use the helmet for cycling. | 0 | 1 | 2 | 3 | 4 |
| I stop and look at both sides before crossing a corner or intersection. | 0 | 1 | 2 | 3 | 4 |
| I periodically check my bike to avoid any mechanical mishap. | 0 | 1 | 2 | 3 | 4 |
| I try to move at a prudent speed to avoid sudden mishaps or braking. | 0 | 1 | 2 | 3 | 4 |
| When I travel at night, I use the necessary safety equipment (lights, vest and / or reflectors). | 0 | 1 | 2 | 3 | 4 |
| I usually keep a safe distance from other cyclists or vehicles. | 0 | 1 | 2 | 3 | 4 |
| When I use the bike path (or bike-lane), I always use the indicated lane. | 0 | 1 | 2 | 3 | 4 |
| I do stretching and/or conditioning exercises before and after using the bike. | 0 | 1 | 2 | 3 | 4 |
| I avoid circulating under adverse weather conditions. | 0 | 1 | 2 | 3 | 4 |
| I avoid circulating if I feel very tired or sick. | 0 | 1 | 2 | 3 | 4 |
| I give way to other vehicles that come very close to me, although I might have the priority. | 0 | 1 | 2 | 3 | 4 |
| I indicate to the other vehicles that I will turn, well in advance. | 0 | 1 | 2 | 3 | 4 |

**II. Cyclist Risk Perception and Regulation Scale**

Please indicate your level of agreement with the following statements, regarding your cycling experience, using this scale:

**Risk Perception**

0 = Strongly disagree; 1 = Disagree; 2 = Neither agree nor disagree; 3 = Agree; 4 = Strongly agree

| I understand the potential consequences of being involved in a traffic accident, for example, with another vehicle | 0 | 1 | 2 | 3 | 4 |
| --- | --- | --- | --- | --- | --- |
| I perceive potentially higher risks for my integrity when I ride a bicycle, than when I am on board of a motorized vehicle | 0 | 1 | 2 | 3 | 4 |
| I am aware of the other vehicles that surround me on the road | 0 | 1 | 2 | 3 | 4 |
| I realize that there are signaling and infrastructure problems that can affect my safety | 0 | 1 | 2 | 3 | 4 |
| I believe that driving under the influence of certain substances (alcohol, illegal and / or prescribed drugs) affects my ability to ride well | 0 | 1 | 2 | 3 | 4 |
| I am aware of the risks involved in using headphones and cellphones while I ride the bicycle | 0 | 1 | 2 | 3 | 4 |
| Riding in urban areas is especially risky, considering the number of vehicles and the complexity of the roads | 0 | 1 | 2 | 3 | 4 |

**Self-reported Rule Knowledge**

| I readily recognize traffic signals | 0 | 1 | 2 | 3 | 4 |
| --- | --- | --- | --- | --- | --- |
| I know the basic rules governing other types of vehicles | 0 | 1 | 2 | 3 | 4 |
| I believe that pedestrians should always have the priority, even with respect to cyclists | 0 | 1 | 2 | 3 | 4 |
| I easily identify areas prohibited to traffic or bicycle parking | 0 | 1 | 2 | 3 | 4 |
| I know the bicycle safety regulations of my city | 0 | 1 | 2 | 3 | 4 |

**III. Cycling distractions**

Normally, do these factors distract you and/or impair your cycling performance during your journeys?

| **Potential distracting source** | **Yes** | **No** |
| --- | --- | --- |
| 01. Text messages or chats |  |  |
| 02. Phone calls |  |  |
| 03. Billboards |  |  |
| 04. People that I find attractive |  |  |
| 05. My own thoughts or concerns |  |  |
| 06. Weather conditions |  |  |
| 07. The behavior of other users of the road |  |  |
| 08. The obstacles in the way |  |  |

**IV. Using-related factors and crash history**

**5.** **I use the bicycle for the following purposes** (You can mark more than one)

☐ Moving regularly to / from school or workplace

☐ Make a short trip to a specific point of the city

☐ Recreation (taking a walk)

☐ Exercise / fitness

☐ As a mean of working

**6. In a week, approximately how many hours do you use the bicycle?**

About _______ hours per week

**7.** **What type of bicycle (s) do you usually use?** (You can mark more than one)

☐ Public bicycles

☐ My own bicycle

☐ Bicycle of a friend or relative

☐ Bicycle provided by my job or company

☐ Other: _________________________

**9.** **In the last 5 years, have you had any accident while riding a bicycle?**

Yes ☐               Do not ☐

***9.1 If you answered "YES" to the previous question*:**

Regardless of their severity, how **many** cycling **accidents have you had** in the last 5 years? ____ accidents.

**10.** **List 3** **reasons - advantages for which you use / prefer the bicycle.**

_____________________________, _____________________________, ________________________________

**11. Finally, list 3 reasons -** **disadvantages for which you** **would think about** **NOT** **using** **the bicycle.**

_____________________________, _____________________________, ________________________________
